# Pregnancy following homologous prepubertal ovarian transplantation in the dog

**DOI:** 10.1186/1743-1050-5-1

**Published:** 2008-04-22

**Authors:** Jennifer K Pullium, Ross Milner, Gary A Tuma

**Affiliations:** 1 Department of Pathology and Laboratory Medicine, Emory University School of Medicine, Atlanta, USA; 2 Center for Comparative Medicine and Surgery, Mount Sinai School of Medicine, New York, USA; 3 Department of Surgery, Division of Vascular Surgery, Emory University School of Medicine, Atlanta, USA; 4 Department of Surgery, Division of Plastic Surgery, Emory University School of Medicine, Atlanta, USA

## Abstract

In several canine models of hereditary human disease the homozygote dogs die prior to puberty, or have substantially reduced fertility. To create a clinically healthy animal that can be bred, but can also transmit the gene of interest, a model of homologous ovarian transplantation in prepubertal dogs was developed. Six dog leukocyte antigen (DLA) identical littermates underwent transplantation of ovarian cortical strips (n = 2) or the entire ovary (n = 4). Immunosuppression was maintained with cyclosporine and MMF in the immediate post-operative period and cyclosporine alone thereafter. All 6 dogs entered puberty and normal semiannual estrus cycles as demonstrated by both physical changes and increasing serum progesterone. Four dogs were bred to a proven stud male, and one became pregnant. Three viable fetuses with observable heartbeats were detected on ultrasound examination. Although the dog eventually aborted the litter, this work represents the first pregnancy achieved following a prepubertal ovarian transplant in the dog.

## Findings

Animal models are critical to the study of inherited human disease. As a result, there has been an enormous increase in research involving genetically engineered mice as models of inherited human disease; however canine models are still utilized throughout the biomedical community. Dogs have advantages over some currently popular rodent models, such as their relatively large size, enabling investigators to more easily implement surgical techniques and collect substantially larger amounts of tissues for analysis. In addition, there are several canine models of hereditary human diseases for which there is no equivalent mouse model.

Since research effort usually revolves around affected homozygotes, the breeding of dogs used as disease models is a major focus of concern. In several models, affected homozygote dogs die prior to puberty or are unable to reliably carry a litter to term due to the effects of their inherited disease and therefore, cannot be bred to produce additional homozygotes. Production of additional homozygotes is thus limited to mating heterozygotes which on average will result in only 25% of offspring carrying the homozygote genotype. Lack of sufficient numbers of affected homozygotes can result in substantial research study delays for investigators that rely on these animals. In addition, maintaining large canine breeding colonies in order to produce sufficient numbers of homozygotes for study can result in prohibitively expensive per diem costs.

Homologous prepubertal gonad transplantation from a mutant donor to a normal littermate is one solution to these problems because this transplantation would allow a normal recipient to be bred, while passing on the genotype of the affected homozygote donor. In this way, the number of homozygotes produced can be increased by 100% over traditional heterozygote breeding.

We reported the initial case of successful prepubertal testes transplantation in the dog [1]. This animal remained fertile seven years postoperatively and sired multiple litters. Successful prepubertal ovarian transplantation would enable the production of litters consisting entirely of affected homozygotes if mated with a testis transplant recipient and an increase in homozygote production of 400% over traditional heterozygote breeding. While ovarian transplantation may initially appear to be a rather extreme method of maintaining the mutant genotype, reproductive physiology of the female dog is very different from that of other domestic species, such as mice and cattle. Since dogs do not ovulate mature oocytes, in vitro maturation would be required before in vitro fertilization could even be attempted, however, these methods have never been successfully (defined by the birth of pups) performed in dogs [2]. Techniques such as superovulation and embryo transfer have also not been performed successfully in dogs [2]. While allogenic orthotopic ovarian transplantation has resulted in ovarian function in humans [3], thus far only monozygotic twins have had successful pregnancies [4,5]. In the present study, we evaluated a whole organ transplant technique that had been successful in mice [6,7] as well as a method transplanting only ovarian cortical strips that had been successful in sheep [8,9].

Each pair of donor and recipient dogs (n = 6; 3 pairs) consisted of prepubertal hound littermates (Covance Research Products, Denver, PA). DLA typing was performed by Midwest Animal Blood Services, Stockbridge, MI, and all dogs receiving transplants were DLA identical. Animals were housed in an AAALAC-accredited facility and all procedures were approved by the Institutional Animal Care and Use Committee. Dogs were anesthetized with 5 mg/kg IV propofol (Diprivan^®^, Zeneca Pharmaceuticals, London UK), maintained on isoflurane (Abbott Laboratories, North Chicago, IL), and prepped as for an ovariohysterectomy. Both donor ovaries were transplanted using one of the following techniques. The en bloc method (n = 4) consisted of removing each recipient ovary in its entirety and placing the donor ovary into the ovarian bursa of the recipient. The bursas were then closed with 7-0 nylon. With the cortical strip method (n = 2), both of the recipient ovaries were removed (leaving only a 5 mm × 5 mm stump of medulla), the cortex of each donor ovary was cut into 1 mm thick strips and placed on the medulla of the recipient ovary. The ovarian bursa of each ovary was then closed with 7-0 nylon.

All dogs were maintained on mycophenolate mofetil (250 mg PO BID 21 days; CellCept^®^, Hoffman-LaRoche Inc, Nutley, NJ) and cyclosporine (15 mg/kg PO q 24 h; Sandimmune^®^, Novartis Pharma AG, Basel, Switzerland) continuously. Following surgery, dogs were monitored daily for signs of graft-versus-host disease and secondary infections as a result of immunosuppressive therapy. In addition, animals were monitored for gingival hyperplasia due to cyclosporine therapy. The transplant recipients were examined every other day for the presence of proestrus vaginal discharge and vulvar swelling. Once signs of vulvar swelling and proestrus bleeding were observed, serum progesterone levels were measured via radioimmunoassay (Yerkes National Primate Research Center Assay Services, Atlanta, GA) in order to determine the appropriate time for mating. When estrus was detected in a timely way, i.e. close to the LH surge, dogs were bred to a proven stud male. Breeding began four days after the initial increase in serum progesterone and was continued every second day until the female would no longer stand for the male. The ovaries of all dogs that were bred were examined by ultrasound examination for the presence of follicles as progesterone began to rise. Approximately 21 days after the first mating, the dogs were examined for the presence of embryos by ultrasound examination. Following post-mortem examination, ovaries were fixed in 10% formalin and sections were stained with hemotoxylin and eosin.

None of the dogs exhibited evidence of graft-versus-host disease. Two dogs demonstrated gingival hyperplasia and were successfully treated with azithromycin (40 mg/kg/day for 5 days). Following surgery, all 6 dogs subsequently demonstrated proestrus vaginal bleeding and vulvar swelling, indicating impending estrus. The LH surge is presumed to occur four days after the progesterone level reaches 0.7 ng/ml [10]. Breeding to a proven fertile male began on the estimated day of the LH surge and continued every other day until the female would no longer stand for the male, marking the end of the estrus period. Four of the six ovary transplant recipients were bred (n = 2 en bloc method; n = 2 cortical strip method), while for the remaining two (en bloc method), behavioral estrus was not detected in a timely way. The latter dogs exhibited serum progesterone levels above 5 ng/ml, indicating that the ovaries were functional but that the dogs were beyond estrus.

**Figure 1:**
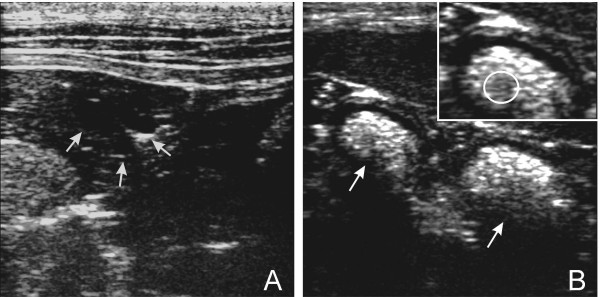
**Ultrasound of follicles on ovary and embryos in uterine horn**. (A) Ultrasound of an ovary approaching estrus in a dog that underwent an en bloc ovarian transplant. Arrows demonstrate typical anechoic follicles, which were also observed in animals having received cortical strip transplants. (B) Ultrasound of uterine horn 28 days after first mating in a dog that received an en bloc ovarian transplant, indicating two of the three embryos found (arrows). The heartbeat was detected in the region outlined by a circle (inset).

Nearing the time of estrus, all four dogs that were bred had multiple follicles on each ovary, visible on ultrasound examination. An example of the ovarian follicles is shown (Figure 1A). One of the four dogs bred (en bloc method) became pregnant. At 28 days after the first mating, three embryos with visible heartbeats (detectable only as a flicker in early gestation) were detected on ultrasound examination (Figure 1B), although the appearance of the embryos was more consistent with 22–23 days gestation [10]. Radiographs at 42 days gestation revealed no fetal skeletons, indicating that the three embryos had been resorbed. Post-mortem examination of the ovaries from the five dogs that did not become pregnant, including the two that were not bred, revealed no patent connection between the ovarian bursa and its respective uterine horn, as demonstrated by the inability to pass dye injected into the uterine tube to the bursa (data not shown). On histology, fibrous connective tissue was found to be occluding the oviduct fimbriae in three of four that received underwent the en bloc method and both animals that received the cortical strips.

The results of this study demonstrate that canine prepubertal ovarian transplantation can result in pregnancy. However, the single case of pregnancy observed in this study terminated in embryo resorption. Although the cause of the resorption is unknown, the embryos appeared underdeveloped for their age, which is common for embryos that will eventually be resorbed [11]. In other species, a decrease in serum progesterone is associated with a loss of early pregnancy, however, progesterone levels in non-pregnant dogs are nearly identical to those found in pregnant dogs as a result of pseudopregnancy [10]. For this reason, serum progesterone was not monitored throughout the pregnancy. Although some have suggested that a lack of progesterone can lead to fetal death, [12,13] there has been no demonstration that insufficient progesterone production can lead to naturally occurring termination of pregnancy in the dog [14].

None of the other three dogs that did not become pregnant (despite normal estrous cycles) rejected the transplanted ovaries or cortical strips, demonstrating that the immunosuppressive therapy was effective. In contrast to our initial experiment with testis transplantation, [1] we chose to use MMF instead of prednisone because corticosteroids have been shown to cause secondary anestrus in females[15]. While the use of cyclosporine has had adverse effects on fertility in rats [16], rabbits that received tubo-ovarian transplants were able to conceive successfully [17]. The effect of cyclosporine on fertility in dogs warrants further investigation. Postmortem evidence suggests that a physical blockage of the oviduct caused by the fibrous connective tissue at the opening of the oviduct fimbriae is a likely explanation for the absence of conception in the dogs that did not become pregnant, although other possibilities such as interference by cyclosporine cannot be excluded. The surgical procedure employed in the present study utilized the natural opening in the ovarian bursa, near the fimbriae, as a site for removal of the recipient ovary and insertion of the donor organ. This natural opening was then sutured closed to secure the ovary, a process that may have resulted in scar tissue formation which obstructed the opening to the oviduct. Avoiding the opening of the ovarian bursa may prevent blockage of the oviduct, which is feasible by approaching the ovary from the opposite side. The dog that became pregnant had undergone the en bloc method of transplantation, demonstrating that this simpler and less time-consuming technique (0.5 hour surgery time) can be used instead of the cortical strip method, which requires a tedious dissection of the ovary (2.5 hours surgery time).

Previous data have demonstrated that prepubertal testes transplantation can be successful in dogs, and the success of prepubertal ovarian transplants would be an additional helpful resource for investigators managing colonies of genetically mutant dogs. This work represents the first pregnancy achieved following a prepubertal ovarian transplant in the dog.

## List of abbreviations

DLA: dog leukocyte antigen; LH: luteinizing hormone.

## Competing interests

The authors declare that they have no competing interests.

## Authors' contributions

JKP, RM, and GAT conceived, designed, and performed the experiments. JKP interpreted the results and wrote the paper. All authors read and approved the final manuscript.

## References

[B1] Pullium JK, Lin PH, Pinter MJ (2001). Homologous transplantation of prepubertal testes in the dog. Transplantation.

[B2] Hewitt DA, England GC (2001). Manipulation of canine fertility using in vitro culture techniques. J Reprod Fertil Suppl.

[B3] Donnez J, Dolmans MM, Pirard C, Van Langendonckt A, Demylle D, Jadoul P, Squifflet J (2007). Allograft of ovarian cortex between two genetically non-identical sisters: case report. Hum Reprod.

[B4] Silber SJ, Gosden RG (2007). Ovarian transplantation in a series of monozygotic twins discordant for ovarian failure. N Engl J Med.

[B5] Silber SJ, Lenahan KM, Levine DJ, Pineda JA, Gorman KS, Friez MJ, Crawford EC, Gosden RG (2005). Ovarian transplantation between monozygotic twins discordant for premature ovarian failure. N Engl J Med.

[B6] Stevens LC (1957). A modification of Robertson's technique of homoiotopic ovarian transplantation in mice. Transplant Bull.

[B7] Cunliffe-Beamer TL, Foster HL, Small JD, Fox JG (1983). Biomethodology and surgical techniques. The Mouse in Biomedical Research.

[B8] Gosden RG, Baird DT, Wade JC, Webb R (1994). Restoration of fertility to oophorectomized sheep by ovarian autografts stored at -196 degrees C. Hum Reprod.

[B9] Baird DT, Campbell B, de Souza C, Telfer E (2004). Long-term ovarian function in sheep after ovariectomy and autotransplantation of cryopreserved cortical strips. Eur J Obstet Gynecol Reprod Biol.

[B10] England GCW (1998). Allen's Fertility and Obstetrics in the Dog.

[B11] England GC, Russo M (2006). Ultrasonographic characteristics of early pregnancy failure in bitches. Theriogenology.

[B12] Davidson AP, Feldman EC, Ettinger SJ, Feldman EC (1995). Ovarian and estrous cycle abnormalities in the bitch. Textbook of Veterinary Internal Medicine.

[B13] Purswell BJ (1992). Differential diagnosis of canine abortion. Curr Vet Ther Small Anim Pract.

[B14] Johnston SD, Root Kustritz MV, Olson PNS (2001). Canine and Feline Theriogenology.

[B15] Kemppainen RJ, Thompson FN, Lorenz MD, Munnell JF, Chakraborty PK (1983). Effects of prednisone on thyroid and gonadal endocrine function in dogs. J Endocrinol.

[B16] Scott JR, Hendrickson M, Lash S, Shelby J (1987). Pregnancy after tubo-ovarian transplantation. Obstet Gynecol.

[B17] Carmona F, Balasch J, Gonzalez-Merlo J (1993). Ovarian function, tubal viability and pregnancy after tubo-ovarian transplantation in the rabbit. Hum Reprod.

